# Urolithin A‐activated autophagy but not mitophagy protects against ischemic neuronal injury by inhibiting ER stress in vitro and in vivo

**DOI:** 10.1111/cns.13136

**Published:** 2019-04-11

**Authors:** Anil Ahsan, Yan‐Rong Zheng, Xiao‐Li Wu, Wei‐Dong Tang, Meng‐Ru Liu, Shi‐Jia Ma, Lei Jiang, Wei‐Wei Hu, Xiang‐Nan Zhang, Zhong Chen

**Affiliations:** ^1^ College of Pharmaceutical Sciences, NHC and CAMS Key Laboratory of Medical Neurobiology Institute of Pharmacology & Toxicology, Zhejiang University Hangzhou China

**Keywords:** autophagy/mitophagy, cerebral ischemia, endoplasmic reticulum stress, neuroprotection, urolithin A

## Abstract

**Aim:**

Mitochondrial autophagy (mitophagy) clears damaged mitochondria and attenuates ischemic neuronal injury. Urolithin A (Uro‐A) activates mitophagy in mammal cells and *Caenorhabditis elegans*. We explored neuroprotection of Uro‐A against ischemic neuronal injury.

**Methods:**

Mice were subjected to middle cerebral artery occlusion. The brain infarct and neurological deficit scores were measured. The N2a cells and primary cultured mice cortical neurons were subjected to oxygen‐glucose deprivation and reperfusion (OGD/R). Uro‐A was incubated during OGD/R, and cell injury was determined by MTT and LDH. Autophagosomes were visualized by transfecting mCherry‐microtubule‐associated protein 1 light chain 3 (LC3). The protein levels of LC3‐II, p62, Translocase Of Inner Mitochondrial Membrane 23 (TIMM23), and cytochrome c oxidase subunit 4 isoform 1 (COX4I1) were detected by Western blot. The ER stress markers, activating transcription factor 6 (ATF6) and C/EBP homologous protein (CHOP), were determined by reverse transcription‐polymerase chain reaction (RT‐PCR).

**Results:**

Urolithin A alleviated OGD/R‐induced injury in N2a cells and neurons and reduced ischemic brain injury in mice. Uro‐A reinforced ischemia‐induced autophagy. Furthermore, Uro‐A‐conferred protection was abolished by 3‐methyladenine, suggesting the requirement of autophagy for neuroprotection. However, mitophagy was not further activated by Uro‐A. Instead, Uro‐A attenuated OGD/R‐induced ER stress, which was abolished by 3‐methyladenosine. Additionally, neuroprotection was reversed by ER stress inducer.

**Conclusion:**

Urolithin A protected against ischemic neuronal injury by reinforcing autophagy rather than mitophagy. Autophagy activation by Uro‐A attenuated ischemic neuronal death by suppressing ER stress.

## 1 INTRODUCTION

Ischemic stroke is one of the leading causes of death and disability worldwide.^1^ The pathological mechanisms underlying ischemic neuronal injury remain not fully elucidated and thus attribute to the shortage of effective pharmacological approaches for stroke therapy.^2^, ^3^

Autophagy is an intracellular catabolic process that turnover of protein and organelles in lysosomes.^4^ Autophagy is activated in ischemic neurons. Several lines of evidence indicated that autophagy protects against ischemic neuronal injury.^5^, ^6^ Autophagy eliminates damaged mitochondria (or terms mitophagy) and subsequently prevents mitochondria‐dependent cell apoptosis and attenuates ischemia/reperfusion‐induced brain injury.^8^, ^9^ Conversely, reinforced mitophagy shows additional neuroprotection against ischemic injury.^10^, ^11^ These findings indicate that mitophagy activation may hold promise as a potential therapeutic strategy against ischemic brain injury.

Urolithins are naturally occurring polyphenols that are produced by human gut microbiota from ellagitannins and ellagic acid. Previous studies have shown that urolithins have multiple biological activities including antioxidant, anticancer, anti‐inflammatory, and antimicrobial properties.^15^, ^16^ Among other urolithins, urolithin A (Uro‐A) was found to induce autophagy in human colorectal cancer cells and macrophages.^20^, ^21^ More recently, Uro‐A was demonstrated to induce mitophagy in *Caenorhabditis elegans* and murine cell lines.^22^ However, whether Uro‐A might activate mitophagy and confer neuroprotection against ischemic injury has not been tested.

## 2 MATERIALS AND METHODS

### 2.1 Animals

Male C57BL/6 mice weighing 22‐25 g were used. All experiments were approved by and conducted in accordance with the ethical guidelines of the Zhejiang University Animal Experimentation Committee and were in complete compliance with the National Institutes of Health Guide for the Care and Use of Laboratory Animals. Every effort was made to minimize any pain or discomfort, and the minimum number of animals was used.

### 2.2 Middle cerebral artery occlusion model and drug treatments

Mice were anesthetized by inhalation of isoflurane. Focal cerebral ischemia was induced by Middle cerebral artery occlusion (MCAO) as described previously.^23^ See Method S1 for more details.

Uro‐A (Toronto Research Chemical, Cat No. U847000) was dissolved in DMSO (50 mg/mL) and further diluted with saline immediately before intraperitoneal (ip) injections. Mice were given an intraperitoneal injection of 2.5 or 5.0 mg/kg of Uro‐A as previously described,^24^, ^25^ for 24 hours and 1 hour before surgery. Mice were grouped into a sham, sham + Uro‐A, MCAO, and MCAO + Uro‐A groups. Mice in the sham group were injected with the same volume of saline.

### 2.3 Infarct analysis

Infarct volume was determined at 24 hours after surgery by staining with 2, 3, 5‐triphenyltetrazolium hydrochloride (TTC, 0.25%). Infarcted areas were analyzed using Image‐Pro Plus 7.0 and determined by the indirect method, which corrected for edema. The percentage of corrected infarct volume was calculated by dividing the infarct volume by the total contralateral hemispheric volume, and this ratio was then multiplied by 100.^26^

### 2.4 Behavioral measurements

Neurological deficit scores were evaluated at 24 hours of reperfusion as followed: 0, no deficit; 1, flexion of the contralateral forelimb on lifting of the whole animal by the tail; 2, circling to the contralateral side; 3, falling to the contralateral side; and 4, no spontaneous motor activity.

### 2.5 Cell culture and oxygen‐glucose deprivation/reperfusion procedures

The primary cortical neuronal culture was performed as described.^27^ See Method S1 for more details.

Mouse neuroblastoma neuro‐2a (N2a, ATCC^®^ CCL‐131™) cells were routinely cultured in DMEM (Gibco) containing 10% fetal bovine serum (FBS, Gibco), 10 U/mL penicillin, and 10 U/mL streptomycin at 37°C in a humidified atmosphere with 5% CO_2_. N2a cells were differentiated in all experiments as following protocol. Briefly, one day after seeding, the culture medium was replaced with differentiation medium (DM), DMEM without FBS and supplemented with N‐2 supplement (100X Gibco) and cells were further incubated for 24 hours to induce differentiation.

The OGD/R model was employed here to mimic ischemia/reperfusion‐like conditions in vitro as we previously used.^28^ See Method S1 for more details. The 2.5 mmol/L of 3‐MA and 10 µmol/L of chloroquine (CQ) were dissolved in normal culture medium and added to the cells at the onset of reperfusion.

### 2.6 Cell viability determination by MTT assay

To examine the neuroprotective effect of Uro‐A against ischemic injury, differentiated N2a cells (1 × 10^5^ cells/well) and primary neurons (3 × 10^5^ cells/well) were plated in 48‐well plates and pretreated with 3‐30 µmol/L of Uro‐A at a highest concentration of 30 μmol/L, which was nontoxic to the cells as previously reported,^22^, ^29^ for 2 hours and then exposed to OGD 4 hours and 1.5 hours, respectively, and reperfusion for 24 hours in the presence of Uro‐A as previously described.^8^ Cell viability was estimated by using dye 3‐4,5‐dimethyl thiazol‐2‐yl‐2,5‐diphenyltetrazolium bromide (MTT, Sigma). The MTT solution was added into each well (5 mg/mL), and the plates were incubated for 2 hours at 37°C. The viability was then measured by evaluating the absorbance at 570 nm. Normal cells were used as the control group, and the cell viability of the control group was assumed to be 100%.

### 2.7 Cell demise determined by lactate dehydrogenase assay

Previous findings have shown that the activity of LDH from damaged cells is proportional to the number of damaged neurons.^30^, ^31^ After exposure to OGD/R, a 50 µL of medium was removed and the amount of LDH leakage from the cells was determined using the LDH kit (Nanjing Jiancheng Bioengineering Institute Nanjing, China) according to the manufacturer's instructions. The absorbance of the samples was read spectrophotometrically at 450 nm. The results are expressed as the percentage of LDH release relative to the control cells.

### 2.8 Plasmids and transfection

Mito‐green fluorescent protein (GFP) was purchased from (Clontech, 637204). Human MAP1LC3B cDNA was amplified from a human kidney cDNA library (YouBio, G102982) by PCR with the forward primer: 5'‐ TCA GAT CTA TGC CGT CGG AGA AGA CCT ‐3' and the reverse primer: 5'‐ GCG AAT TCT TAC ACT GAC AAT TTC ATC CCG ‐3'. The PCR product was inserted into the BglII and EcoRI site of pmCherry‐C1 plasmid to construct the pmCherry‐LC3 plasmid. Primary cortical neurons were transfected with Mouse Neuron Nucleofector® Kit (Lonza, VPG‐1001) according to the manufacturer's protocol. Briefly, 5 × 10^6^ cells were collected during primary neuronal culture and then resuspended with 100 μL Nucleofector® Solution containing 3 μg DNA. The cell suspension was transferred into a cuvette and electroporated with Nucleofector® Program O‐005.

The N2a cells were transfected using jet PRIME Reagent (Polyplus transfection) at 24 hours before further experiments.

### 2.9 Confocal microscopy and image analysis

For live microscopy examination, N2a cells and primary neurons were seeded onto a poly‐l‐lysine‐treated glass bottom dish (In Vitro Scientific, D35‐20‐0‐N) transfected with mCherry‐LC3 and Mito‐GFP. After OGD, N2a cells and primary neurons were treated with 10 and 30 µmol/L of Uro‐A and re‐perfused for 3 hours. Optical z series images were obtained by using a confocal microscope (Leica Microsystems, Germany). The number and area occupied by LC3 puncta and Mander's overlap efficiency was measured and analyzed as described by Image‐Pro Plus 7.0 software.^32^ For each condition, a total of 50 cells were analyzed.

### 2.10 Quantitative real‐time polymerase chain reaction

To detect the mRNA levels of ATF6 and C/EBP homologous protein (CHOP), the total RNA was isolated from N2a cells after 1 hour of OGD/R with 3‐30 µmol/L of Uro‐A and ischemic brain tissues after 12 hour of MCAO by using ice‐cold Trizol reagent (Sigma, T9424). Total RNA was reverse‐transcribed into cDNA using the Prime Script One‐Step reverse transcription‐polymerase chain reaction (RT‐PCR) Kit (Takara, RR037A). PCR amplifications were performed with Brilliant II SYBR green QPCR master mix (Takara, RR037A). The primer sequences were as follows: mouse CHOP (Fw: 5‐GTCCAGCTGGGAGCTGGAAG‐3; Rev: 5‐CTGGTCAGGCGCTCGATTTCC‐3), mouse ATF6 (Fw: 5‐AAGTATGGGTTCGGATAT‐3; Rev: 5‐CTCTGACACCACCTCGTC‐3), and mouse β‐actin (Fw: 5‐CTGTCCCTGTATGCCTCTG‐3; Rev: 5‐ATGTCACGCACGATTTCC‐3). Beta‐actin was used as the endogenous control. The relative expression value was calculated via the 2^–ΔΔCt^ method.

### 2.11 Western blot analysis

Protein extraction, quantification, and immunoblotting were performed as described previously.^13^ See Method S1 for more details.

### 2.12 Statistical analysis

All the data were performed using Graph Pad Prism 7 and expressed as mean ± SD. One‐way analysis of variance was used to perform statistical analysis. *P* < 0.05 was considered statistically significant.

## 3 RESULTS

### 3.1 Uro‐A inhibited OGD/R‐induced cell injury in N2a cells and primary cultured neurons

**Figure 1:**
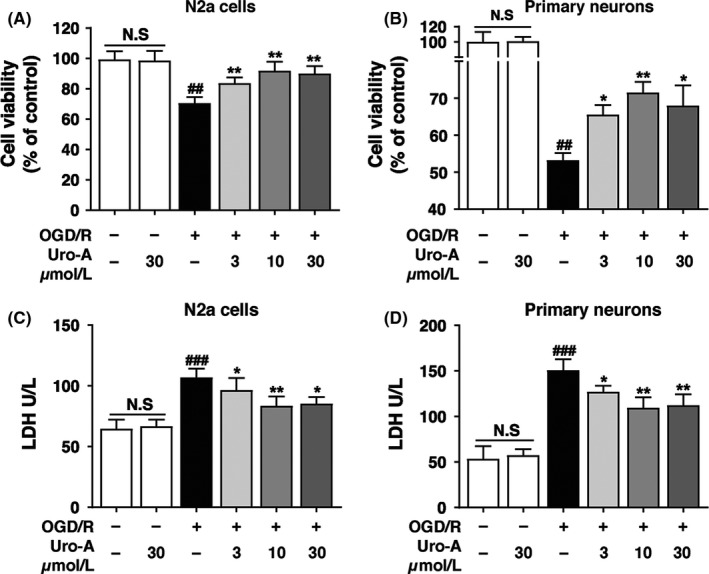
The neuroprotective effect of urolithin A on the viability of N2a cells and primary cultured neurons against OGD/R injury (A, B) Differentiated N2a cells and primary cultured cortical neurons were exposed to OGD for 4 h and 1.5 h, respectively, and treated with 3‐30 µmol/L of Uro‐A for 24 h reperfusion (A) Cell viability of N2a cells and (B) primary cultured cortical neurons was determined by MTT assay (C, D) Differentiated N2a cells and primary cultured cortical neurons were treated as described above, and then, the culture medium was collected to examine lactate dehydrogenase (LDH) activity (C) LDH activity in the culture medium of N2a cells and (D) primary cultured cortical neurons. Differences are significant at *^#^P* < 0.05, *^##^P* < 0.01, *^###^P* < 0.001 vs control groups: *P < 0.05 **P < 0.01 vs OGD/R alone groups, NS, not statistically significant of indicated groups. Data are expressed as mean ± SD from five (n = 5) independent experiments

Cell viability assays (MTT) indicated that OGD/R treatment in both N2a cell and primary cultured neurons dramatically reduced the cell viability to 70 ± 3.9% and 53.2 ± 1.9% of control, respectively, while Uro‐A treatment significantly reversed viability loss dose dependently with a maximal effect of 10 µmol/L of Uro‐A (91.7 ± 6.0% and 71.4 ± 2.9%) of OGD/R (Figure 1A and B). In both intact N2a cells and primary cultured neurons, the reduction of cell viability was not observed at a concentration of 30 µmol/L.

Neuronal damage was also quantitatively assessed by measuring the activity of LDH released from both N2a cells and primary cultured neurons. The relative LDH release increased from 64.7 ± 7.5 U/L‐107 ± 7.1 U/L and 53.5 ± 13.7 U/L‐150.8 ± 12.0 U/L, respectively, after OGD/R, while the administration of Uro‐A significantly decreased the LDH release in a dose‐dependent manner with the maximal effect at 10 µmol/L of Uro‐A (Figure 1C and D). Moreover, treatment with Uro‐A alone at the dose of 30 µmol/L had no significant influences on LDH release. These results indicated that Uro‐A attenuated the OGD/R‐induced neuronal injury.

### 3.2 Uro‐A reinforced ischemic reperfusion‐induced autophagy both in vitro and in vivo

**Figure 2:**
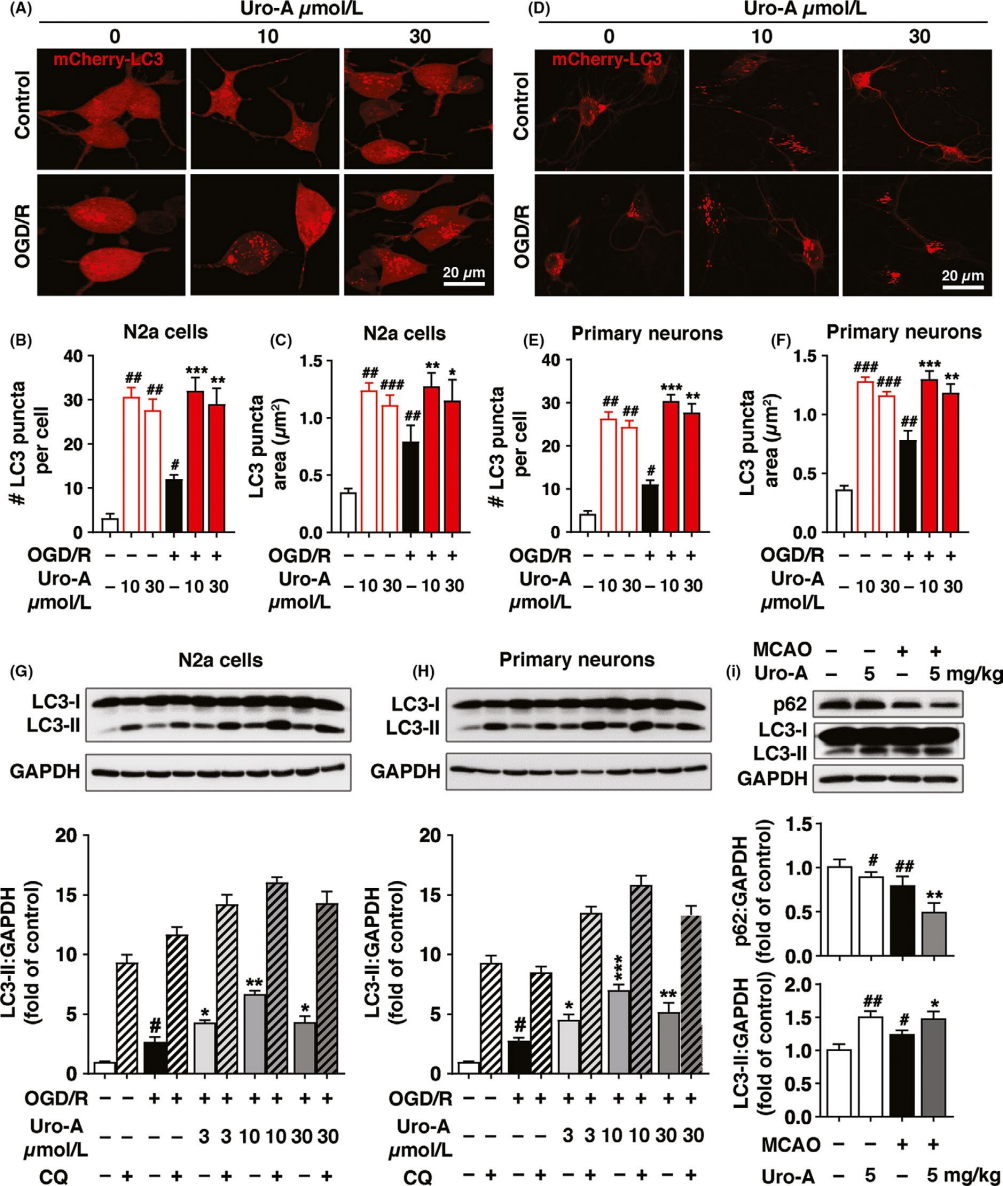
Urolithin A reinforces autophagy in vitro and in vivo after neuronal ischemia. N2a cells and primary cultured neurons were transfected with mCherry‐LC3 and exposed to OGD for 4 h and 1.5 h, respectively, and treated at the concentration of 10 and 30 µmol/L Uro‐A. Images were captured at 3 h after reperfusion by confocal microscopy (A) Fluorescent images of mCherry‐LC3 in N2a cells (B) Columns represent the number of autophagosomes in a single cell (C) Columns represent the area of each LC3 puncta in one N2a cell (D) Representative images of primary cultured neurons (E) Columns represent the number of autophagosomes in a single neuron (F) Columns represent the area of each LC3 puncta in one neuron. At least 50 cells from at least 3 independent experiments were counted in each group (G**)** N2a cells were exposed to OGD for 4 h.(H) Primary cultured neurons were exposed to OGD for 1.5 h and treated with 3‐30 µmol/L of Uro‐A. At 6 h of reperfusion, LC3‐II and GAPDH levels were determined by Western blot. Semi‐quantitative analysis of LC3‐II bands was shown (lower panel) (I) Mice were subjected to MCAO for 1 h. After 12 h of reperfusion, LC3‐II, p62, and GAPDH levels in ischemic core were assessed by Western blot, n = 5 per group. Semi‐quantitative analysis of LC3‐II and p62 bands was shown (lower panel). Differences are significant at *^#^P* < 0.05, *^##^P* < 0.01, ^#^ *^##^P* < 0.001 vs control groups: *P < 0.05 **P < 0.01 ***P < 0.001 vs OGD/R alone groups. *^#^P* < 0.05, *^##^P* < 0.01 *vs*. sham group: *P < 0.05 **P < 0.01 *vs.* MCAO. Data are expressed as mean ± SD values. Scale bar, 20 μm

To examine whether Uro‐A activates autophagy in ischemic neuronal cells, we quantified the LC3‐positive puncta number and size, which were widely used to indicate autophagosome formation.^33^ By transfecting mCherry‐LC3 to N2a cells, we found significantly increased quantity of mCherry‐LC3‐positive puncta in N2a cells after 3 hour of reperfusion, indicating autophagy activation by ischemia, which was in line with previous studies.^8^ Furthermore, treatment with Uro‐A (10 and 30 µmol/L) further increased the puncta number dose dependently. The 10 µmol/L of Uro‐A showed the maximal effect by increasing the puncta number under both physiological from 3.1 ± 1.0 to 30.6 ± 2.0 and OGD/R from 12 ± 1.0 to 32 ± 3.0 conditions (Figure 2A and B). In addition, puncta area was similar in both conditions and increased approximately 1‐fold after OGD/R treatment (Figure 2A and C). We next determined the effect of Uro‐A on autophagy by employing in primary neurons. The results showed that Uro‐A further increased OGD/R‐induced autophagy activation as reflected by an increased number and area of mCherry‐LC3‐positive puncta (Figure 2D‐F). To exclude autophagosome accumulation caused by lysosome dysfunction, we determined the LC3 expression in the presence of lysosome inhibitor CQ in both N2a cells and primary cultured neurons. The results showed that Uro‐A (3‐30 µmol/L) activated the expression of LC3‐II in both intact N2a cells and primary cultured neurons (Figure S1A, B). Notably, Uro‐A reinforced OGD/R‐upregulated LC3‐II, which was further accumulated with the incubation of CQ, supporting that Uro‐A further activated autophagy rather than caused lysosome dysfunction in ischemic neuronal cells (Figure 2G and H).

To identify whether Uro‐A can also activate autophagy in ischemic brain, mice were subjected to MCAO and the expressions of autophagy‐related proteins, including LC3 and p62, in ischemic brains were determined by Western blot at 12 hours after reperfusion. MCAO significantly increased the LC3‐II and reduced the p62 protein level indicating the activation of autophagy. Moreover, pretreatment with Uro‐A (5 mg/kg) enhanced autophagy in both sham and MCAO group, reflecting by accumulation of LC3‐II and decreased the level of p62 (Figure 2I). Collectively, these results suggested that Uro‐A activated autophagy and promoted autophagosome maturation in both ischemic neuronal cells and ischemic brains.

### 3.3 Autophagy is required for Uro‐A conferred neuroprotection

**Figure 3:**
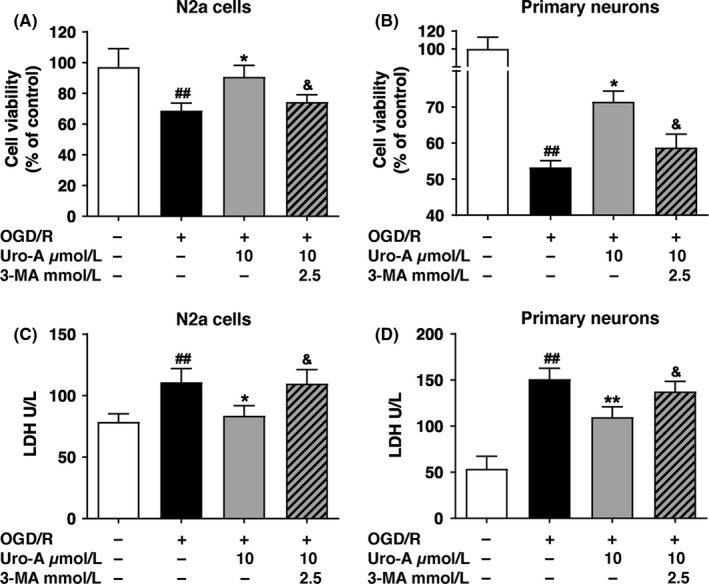
Autophagy is required for urolithin A conferred neuroprotection (A, B) Differentiated N2a cells and primary cultured cortical neurons were exposed to OGD for 4 h and 1.5 h, respectively, and treated with 10 µmol/L of Uro‐A with or without 3‐MA at concentration 2.5 mmol/L for 24 h reperfusion (A) Cell viability of N2a cells and (B) cell viability of primary cultured neurons were measured by MTT assay (C, D) Differentiated N2a cells and primary cultured cortical neurons were treated as described above, and then, the culture medium was collected to examine LDH activity (C) Lactate dehydrogenase (LDH) level in N2a cells and (D) in primary cultured cortical neurons. Differences are significant at*^ ##^P* < 0.01 vs control groups: *P < 0.05, **P < 0.01 *vs.* OGD/R alone groups and *^&^P* < 0.05 vs Uro‐A groups. Data are represented as the mean ± SD from at least five (n = 5) independent experiments

To verify whether autophagy induction is involved in the neuroprotective effect of Uro‐A, we incubated the N2a cells and primary cultured neurons with 10 µmol/L of Uro‐A in the presence or absence of autophagy inhibitor 3‐MA during OGD/R, at which the most intense autophagy flux was observed. We found that co‐incubation of 3‐MA significantly abolished the neuroprotection of Uro‐A by reversing the increase in cell viability both in N2a cells and primary cultured neurons (Figure 3A and B). Inconsistent with the results of cell viability, we found that attenuated LDH leakage with Uro‐A treatment was significantly abolished by 3‐MA (Figure 3C and D). Overall, these data indicated that the autophagy activation was required for the neuroprotective effect of Uro‐A.

### 3.4 Uro‐A does not induce mitophagy both in ischemic neuronal cells and in vivo

**Figure 4:**
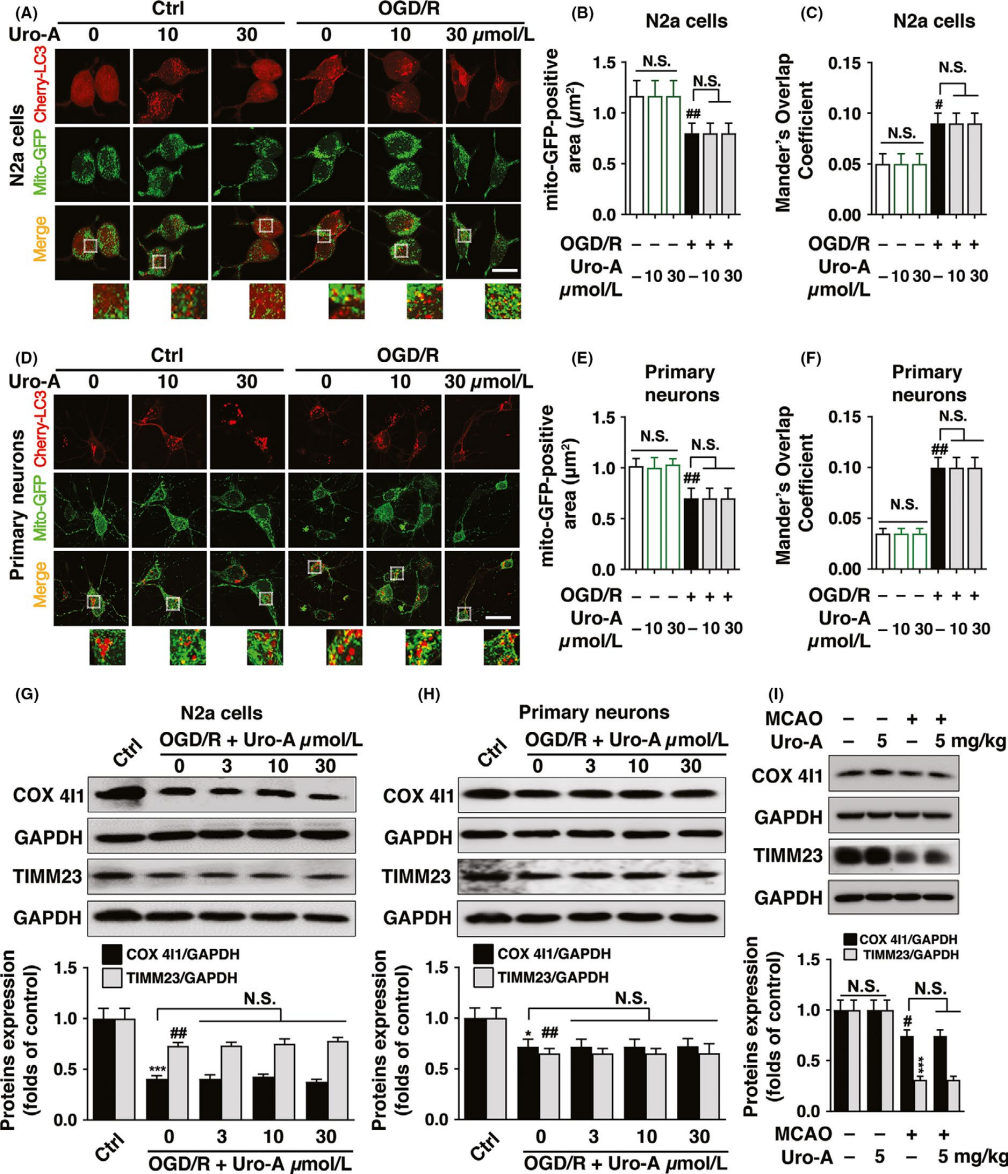
Urolithin A does not induce mitophagy in cerebral ischemia models (A‐F) N2a cells and primary cultured cortical neurons were transfected with mCherry‐LC3 and Mito‐GFP vectors to visualize LC3 puncta and mitochondria, respectively. Both cells were subjected to OGD for 4 h and 1.5 h, respectively, and treated at the concentration of 10 and 30 µmol/L Uro‐A. After 3 h of reperfusion, (A) Representative fluorescent images of N2a cells were captured by confocal microscopy (B) Columns represent the mito‐GFP‐positive area of N2a cells (C) Columns represent Mander's overlap coefficient of mito‐GFP and mCherry‐LC3 of N2a cells (D) Representative images of primary cultured neurons (E) Columns represent the mito‐GFP‐positive area of primary cultured neurons (F) Columns represent Mander's overlap coefficient of mito‐GFP and mCherry‐LC3 of primary cultured neurons. At least 30 cells from three independent experiments for each group were included. TIMM23 and COX4I1 protein levels were determined by Western blot analysis after 6 h of reperfusion treated with 3‐30 µmol/L of Uro‐A (G) in N2a cells and (H) in primary cultured neurons. Semi‐quantitative analysis of TIMM23 and COX4I1 bands was shown (lower panel) (I) Mice were subjected to MCAO for 1 h, and the expression of TIMM23 and COX4I1 in ischemic core was assessed by Western blot after 12 h of reperfusion, n = 5 per group. Semi‐quantitative analysis of TIMM23 and COX4I1 bands was shown (lower panel). Differences are significant at *^#^P* < 0.05, *^##^P* < 0.01, ***P < 0.001 vs control groups. NS, not statistically significant of indicated groups. Data are shown as mean ± SD. Scale bar, 20 μm

Previous research showed that Uro‐A induced mitophagy in mammal cells and *C elegans*.^22^ In addition, our previous studies indicated that autophagy conferred its neuroprotection by promoting mitophagy.^8^ Here we found Uro‐A‐activated autophagy involved in the neuroprotection, and we further asked whether Uro‐A enhanced mitophagy. To this end, we transfected both N2a cells and primary cultured neurons with mito‐GFP and mCherry‐LC3 to visualize mitochondria and autophagosomes, respectively. To our surprise, Uro‐A alone did not induce mitophagy in both intact N2a cells and neurons at the concentration of either 10 or 30 μM, indicated by similar mito‐GFP‐positive area to control group. Consistent with our previous studies, OGD/R significantly reduced the area of mitochondria labeled by mito‐GFP in both N2a cells and neurons, which suggested mitophagy activation.^28^ Nevertheless, 10 and 30 µmol/L of Uro‐A treatment did not further decrease the mitochondrial content, implying mitophagy was not reinforced by Uro‐A in either N2a cells or neurons (Figure 4A, B, D, and E). We next quantified the colocalization of mito‐GFP and mCherry‐LC3 puncta in both N2a cells and neurons. The results showed Uro‐A treatment failed to increase mito‐GFP and mCherry‐LC3 puncta overlap (Figure 4C and F), suggesting Uro‐A did not reinforce mitophagy in ischemic neuronal cells. To further verified this notion, we examined the constitutively expressed mitochondrial markers COX 4I1 and TIMM23 by Western blot. Similarly, Uro‐A treatment did not further reduce the COX 4I1 and TIMM23 in OGD/R‐treated N2a cells and primary cultured neurons (Figure 4G and H). Additionally, Uro‐A failed to reduce mitochondrial proteins COX 4I1 and TIMM23 in both intact N2a cells and primary cultured neurons even at the concentration of 30 µmol/L (Figure S1C, D). We further verified whether Uro‐A induced mitophagy in ischemic brain. MCAO induced the significant loss of mitochondrial proteins COX 4I1 and TIMM23; nonetheless, Uro‐A failed to further reduce mitochondrial proteins COX 4I1 and TIMM23 in ischemic brain (Figure 4I). These data indicated that Uro‐A did not induce mitophagy in both ischemia/reperfusion‐injured neuronal cells and mice.

### 3.5 Uro‐A represses the ER stress by activation of autophagy both in vitro and in vivo

**Figure 5:**
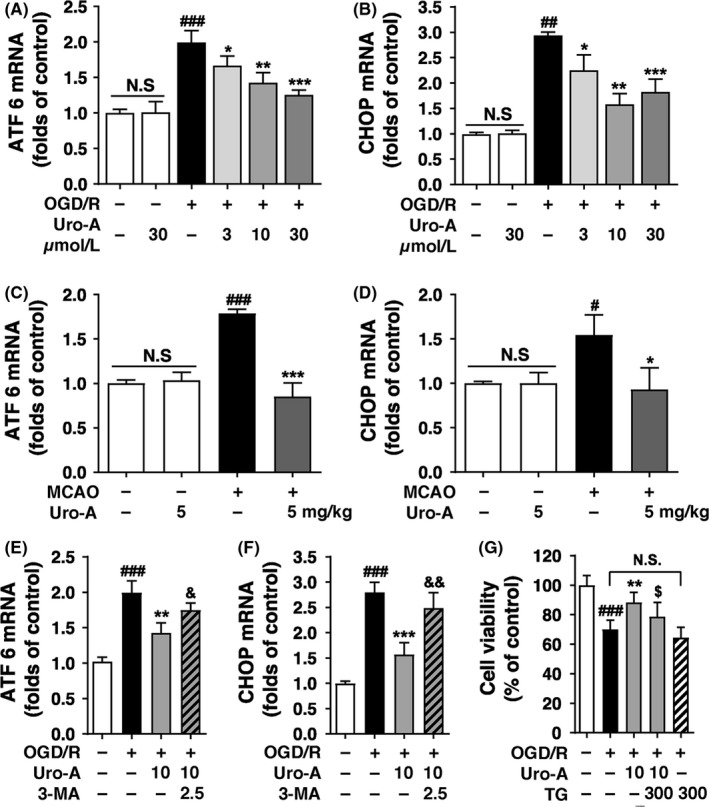
Urolithin A alleviated neuronal ischemic‐induced ER stress (A, B) N2a cells were subjected to OGD for 4 h and treated with 3‐30 µmol/L of Uro‐A for 1‐h reperfusion. Total RNA was collected from N2a cells and used for the RT‐PCR analysis (A) ATF 6 and (B) CHOP (C‐D) The levels of ATF6 and Chop in ischemic core region were measured at 12 h of reperfusion after 1 h of MCAO by RT‐PCR (C) ATF 6 and (D) CHOP (n = 5). The mRNA levels of (E) ATF 6 and (F) CHOP in N2a cells treated with and without 2.5 mmol/L of 3‐MA were normalized to β‐actin (G) Cell viability of N2a cells was measured by MTT after treating with 10 µmol/L of Uro‐A in the presence and absence of thapsigargin at the concentration (300 nmol/L) for 24 h. Differences are significant at *^#^ P* < 0.05 *vs.* sham groups,*^ ##^P* < 0.01, *^###^P* < 0.001 vs control groups, *P < 0.05 vs MCAO, **P < 0.01, ***P < 0.001 vs OGD/R alone groups, *^&^P* < 0.05, *^&&^P* < 0.01 vs Uro‐A groups, and *^$^P* < 0.05 vs TG group. NS, not statistically significant of indicated groups. Data are expressed as mean ± SD, n = 5 per group.

Endoplasmic reticulum stress was activated in ischemic brains and resulted in neuronal demise.^34^ As a consequence, autophagy helps to alleviate ER stress.^35^, ^36^ We thus hypothesize that Uro‐A might suppress ER stress by activating autophagy. We determined mRNA levels of ATF6 and CHOP both in vitro and in vivo, which reflected the extension of ER stress.^37^, ^38^ Our data indicated that both ATF6 and CHOP were significantly increased after OGD/R Of note, treatment with Uro‐A (3‐30 µmol/L) significantly decreased OGD/R‐induced upregulations of ATF6 and CHOP in a dose‐dependent manner (Figure 5A and B). Consistent with the changes in ER stress in N2a cells*,* quantitative PCR confirmed the upregulation of ATF6 and CHOP after MCAO both which were substantially inhibited by Uro‐A (Figure 5C and D). These data suggested that Uro‐A alleviated ischemia‐induced ER stress both in vitro and in vivo.

To confirm the involvement of autophagy in ER stress alleviation with Uro‐A treatment, we blocked autophagy with 3‐MA. The results showed that compared with Uro‐A treatment alone, 3‐MA significantly reversed the mRNA levels of ATF6 and CHOP, suggesting autophagy was involved in alleviating ER stress by Uro‐A in OGD/R‐treated neuronal cells (Figure 5E and F). Furthermore, we examined whether ER stress alleviation contributed to the neuroprotective effect of Uro‐A against ischemia. To this end, an ER stress activator, thapsigargin, was added to N2a cells alone or with Uro‐A during reperfusion. We found thapsigargin significantly abolished the neuroprotection of Uro‐A as reflected by a remarkable reduction of cell viability. Thapsigargin alone showed no impact on cell viability, which excluded the cytotoxicity in this concentration (Figure 5G). Taken together, these results indicated that Uro‐A alleviated OGD/R‐induced ER stress by activating autophagy and thus protected neuronal cells from ischemic injury.

### 3.6 Uro‐A reduced acute ischemic brain injury in mice

**Figure 6:**
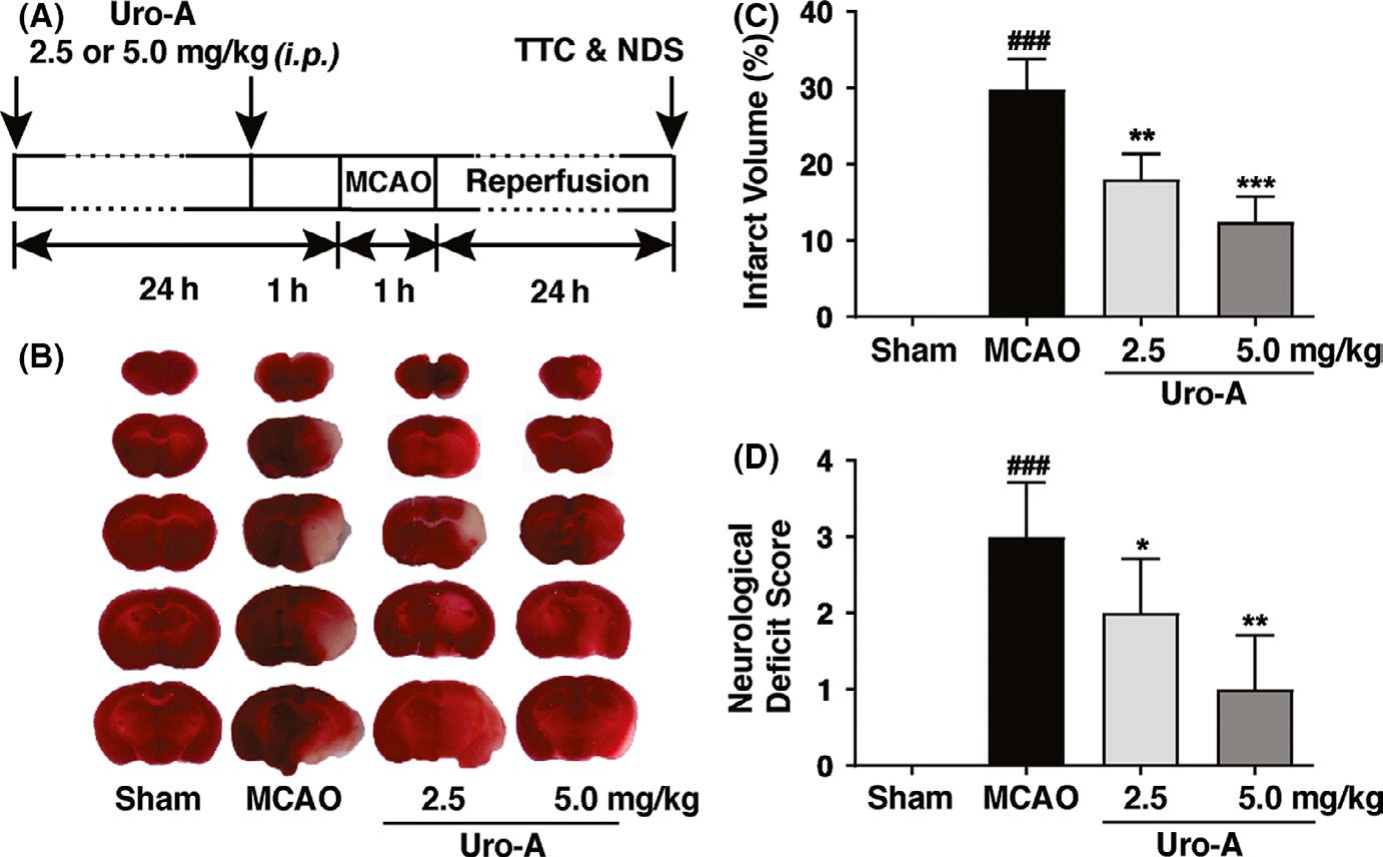
Urolithin A reduced ischemic brain injury in MCAO mice (A) The schematic experimental protocol for in vivo model, mice were administrated with 2.5 and 5.0 mg/kg of Uro‐A (intraperitoneal) twice before MCAO surgery and subjected to MCAO for 1 h followed by 24‐h reperfusion (B) Representative TTC‐stained brain slices after 24 h of MCAO are shown (C) The infarct volume between vehicle and Uro‐A (2.5 or 5.0 mg/kg)‐treated MCAO mice was expressed as the ratio of (infarct volume/the whole brain volume) ×100%. The white area defined the infarct area (D) Neurological deficit scores (NDS) were measured at 24 h after surgery. Differences are significant at *^###^P* < 0.001 vs sham group, *P < 0.05 and **P < 0.01 vs MCAO group. Data are presented as mean ± SD from seven (n = 7) mice in each group

The neuroprotective effect of Uro‐A against ischemia was further investigated in vivo by using a temporary middle cerebral artery occlusion (MCAO) model in mice. The results showed that MCAO induced 29.8 ± 3.9% of infarct volumes and 3.0 ± 0.7% of neurological deficit score in mice. The 2.5 and 5.0 mg/kg of Uro‐A treatments significantly decreased the volume of infarction to 18 ± 3.3% and 12.4 ± 3.3%, respectively. In line with that, the neurological deficit score reduced to 2.0 ± 0.7 and 1.0 ± 0.5, respectively (Figure 6B‐D). The data indicated that Uro‐A treatment protects against ischemic brain injury.

## 4 DISCUSSION

Effective therapeutic intervention for ischemic stroke remains a challenge. Uro‐A can cross the blood‐brain barrier and has been shown to protect neuronal cell in the context of Alzheimer's disease.^39^ Emerging data indicated Uro‐A attenuated ischemic injury in myocardial cells.^24^ Nevertheless, whether Uro‐A may protect against cerebral ischemia remains undetermined. By employing the OGD/R model, we found Uro‐A significantly reduced ischemic neuronal injury, either in differentiated N2a cells or in primary cultured neurons (Figure 1). More importantly, the systemic administration of Uro‐A showed a prominent effect in reducing focal brain ischemia in mice (Figure 6B and C). This neuroprotection was accompanied by improved neurological deficit score (Figure 6D). Therefore, the present study provided evidence, for the first time, indicating the neuroprotective effect of Uro‐A against acute ischemic injury. We found the dosage of 2.5 and 5 mg/kg of Uro‐A pretreatment was sufficient to reduce ischemic brain injury. Since the availability of Uro‐A in diet, we propose that constant Uro‐A consumption may be beneficial for the individuals who suffer from stroke risks.

Although the benefits of autophagy in acute ischemic brains have been documented,^8^, ^9^ few compounds were known to rescue ischemic brain injury by targeting autophagy.^40^, ^41^ Uro‐A activates autophagy in several types of cell,^20^, ^22^ yet it has been undetermined whether Uro‐A also activates autophagy in ischemic neuronal cells and mice brain. Here we clearly showed that Uro‐A increased both the number and size of autophagosomes in ischemic neuronal cells (Figure 2A‐F) and autophagy inhibition significantly abolished the neuroprotection of Uro‐A (Figure 3). These shreds of evidence brought forth a novel role of Uro‐A in protecting against ischemic brain injury by activating autophagy. Uro‐A showed multiple biological activities in a variety type of cell. In particular, Uro‐A inhibited neuroinflammation and reduced oxidative stress in neuronal cells,^29^, ^42^ which were highly relevant to ischemic neuronal injury. Autophagy suppresses neuroinflammation 43 and attenuates oxidative stress 44 in ischemic brains. Hence, it is likely that autophagy activation is the primary mechanism underlying the benefits of Uro‐A. It should be noted that it remains unclear how autophagy may contribute to chronic cerebral ischemia. Hence, we did not test the potential neuroprotection of Uro‐A in chronic ischemic model in the present study, which should be addressed by further studies.

Autophagy protected against ischemic injury by inducing mitophagy.^8^ In addition, Uro‐A induced mitophagy in mammal cells and *C elegans*.^22^ We thus hypothesized Uro‐A may reduce ischemic neuronal injury by reinforcing mitophagy. However, we did not find mitochondrial loss with Uro‐A alone in intact cells nor in OGD/R‐treated neuronal cells or in ischemic brain tissues (Figure 4). Accordingly, 10 µmol/L of Uro‐A treatment failed to increase the overlap of autophagosomes with mitochondria, even though autophagosome number was increased in this concentration (Figure 4A and D). Therefore, we concluded that Uro‐A did not activate mitophagy in ischemic neurons despite that autophagy can be activated. Uro‐A induced mitochondrial membrane potential (ΔΨm) loss which further activated mitophagy in a PINK1/Parkin‐dependent pathway in muscle cells.^22^ However, compared with non‐neuronal cells, Parkin‐mediated mitophagy takes place in a delayed manner in neurons,^45^ which might account for little mitophagy activation in intact neurons in the observed time scale. Furthermore, in ischemia/reperfusion‐treated neurons, massive ΔΨm loss occurred and subsequently led to extensive mitophagy (Figure 4).^9^, ^13^ It thus can be speculated that Uro‐A failed to increase the extension of mitophagy in ischemic neurons and in vivo because minimum ΔΨm loss can be further activated by Uro‐A. In addition, Uro‐A also failed to induce mitochondrial loss in aged cells.^22^ Here we provided evidence doubting the causality of Uro‐A on mitophagy induction. Interestingly, Uro‐A still induced autophagy regardless of mitophagy inactivation. This phenotype implied that Uro‐A may induce autophagy and mitophagy in separate signaling cascades, which need to be addressed in future studies. Our further investigation suggested that Uro‐A relieved OGD/R‐induced ER stress (Figure 5), which was reversed by autophagy inhibition (Figure 5E and F). These results were in line with the established role of autophagy in suppressing ER stress.^46^, ^47^ Our previous finding suggested that moderate ER stress during ischemia‐reperfusion enhanced mitophagy process by targeting autophagosomes to damaged mitochondria.^35^ It is plausible that Uro‐A might suppress ER stress induced by OGD/R and MCAO and thus attenuate mitophagy reinforcement. Furthermore, we found that ER stress suppression could be a critical mechanism underlying the neuroprotective effect of Uro‐A, since thapsigargin, an ER stress inducer, abolished the neuroprotection of Uro‐A (Figure 5G). These observations may make the first link of ER stress with Uro‐A‐induced autophagy in ischemic neurons.

Taken together, our data advocated the neuroprotective effects of Uro‐A against ischemic injury by activating autophagy. Although it was documented that Uro‐A induced both autophagy and mitophagy, Uro‐A failed to induce mitophagy in ischemic neuronal cells and brains. Alternatively, Uro‐A suppressed ER stress by inducing autophagy and prevented neuronal death. Our work provides new insights into the neuroprotective properties of Uro‐A, which may give a pharmacological basis for Uro‐A as a promising compound for the treatment of cerebral ischemic stroke.

## CONFLICT OF INTEREST

The authors have no conflict of interest.
