# Supplementary figures and images for: Urolithin A‐activated autophagy but not mitophagy protects against ischemic neuronal injury by inhibiting ER stress in vitro and in vivo
Source: CNS Neurosci Ther. 2019 Apr 11;25(9):976–86. doi: 10.1111/cns.13136 (PMC6698978; doi:10.1111/cns.13136)

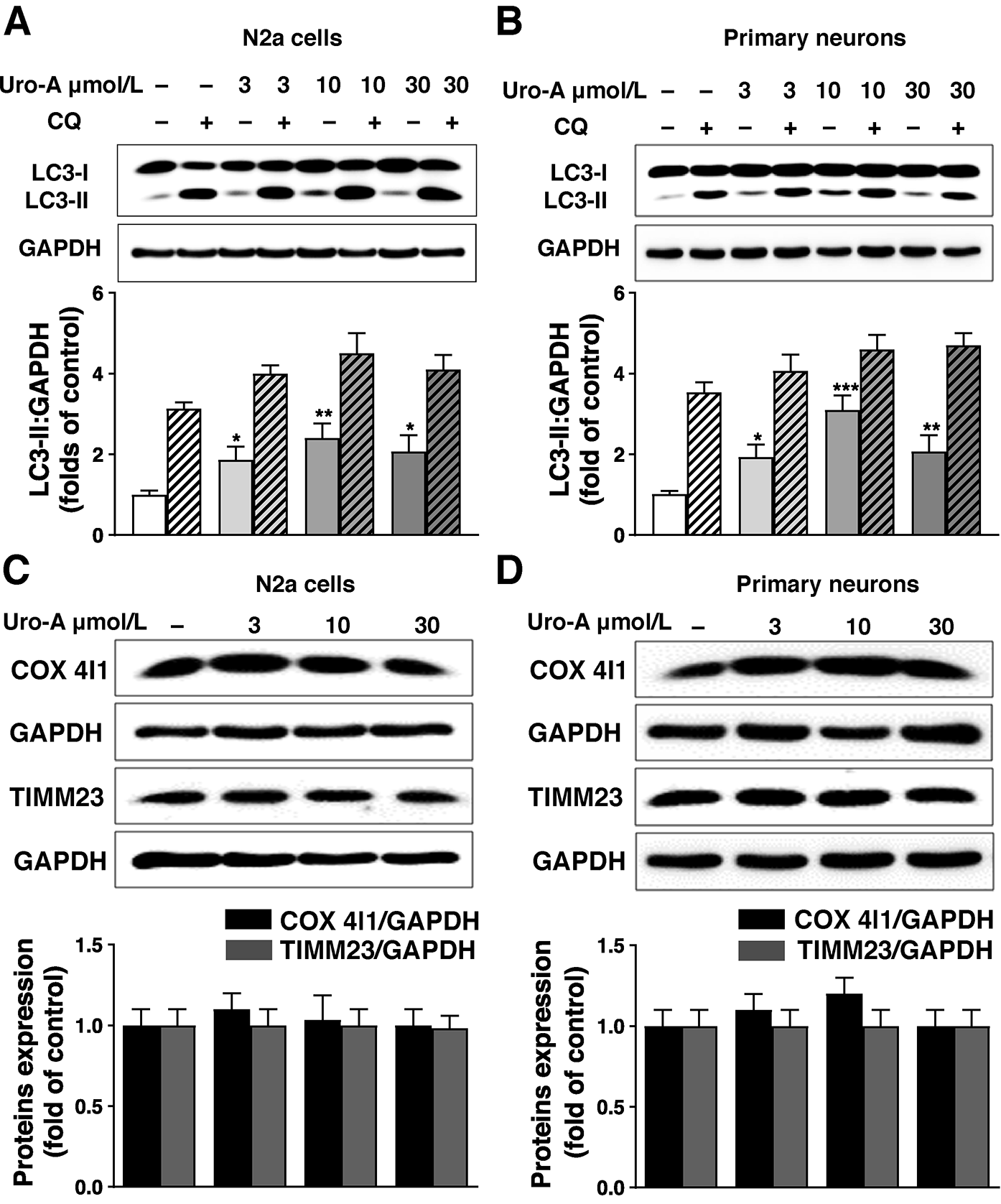

Supplement: Supplementary file 1 [file CNS-25-976-s001.tif]
