# Supplementary material for: Urolithin A‐activated autophagy but not mitophagy protects against ischemic neuronal injury by inhibiting ER stress in vitro and in vivo
Source: CNS Neurosci Ther. 2019 Apr 11;25(9):976–86. doi: 10.1111/cns.13136 (PMC6698978; doi:10.1111/cns.13136)
Supplement: Supplementary file 2 [file CNS-25-976-s002.docx]

**Supplemental methods**

**Middle cerebral artery occlusion (MCAO) model**

Mice were anesthetized with 4% isoﬂurane with a mixture of 60% medical air and 40% oxygen; anesthesia was maintained with 2% isoﬂurane. Cerebral blood flow (CBF) was determined in the area of the middle cerebral artery (MCA) by laser Doppler flowmetry (Moor Instruments, Devon, UK). A flexible fiber-optic probe was affixed to the skull over the cortex supplied by the proximal part of the right MCA (2 mm caudal to bregma and 6 mm lateral to midline). Animals with < 80% reduction in CBF in the core of the MCA area were excluded from the study. Focal cerebral ischemia was induced by MCA occlusion (MCAO). Briefly, a 6-0 nylon monofilament sutures, blunted at the tip and coated with 1% poly-L-lysine, was inserted 10 mm into the internal carotid to occlude the origin of the MCA. Reperfusion was allowed after 60 min by monofilament removal. Body temperature was maintained at 37 °C by a heat lamp (FHC, Bowdoinham) during surgery and for 2 h after the start of reperfusion. Mice in sham group experienced the same surgery process without suture occlusion.

**Primary cortical neuronal culture**

For primary cortical neuronal culture, E18 mice were used. Briefly, the dissected cortex was treated with 0.125% trypsin in Hank’s buffer (in mmol/L: 137 NaCl, 5.4 KCl, 0.4 KH2PO4, 0.34 Na2PO4·7H2O, 10 glucose and 10 HEPES) for 30 min at 37 °C and dissociated by repeated passage through a series of fire-polished Pasteur pipettes. Approximately 2 × 10^5^ cells/cm2 were seeded onto poly-Llysine (10 mg/ml)-coated plates and dishes. The neurons were grown in Neurobasal medium (Invitrogen, 21103–049) supplemented with 2% B27 (Invitrogen, 17504–044), 10 U/ml penicillin, 10 U/ml streptomycin, and 0.5 mmol/L glutamine at 37 °C in a humidified atmosphere with 5% CO_2_. Cultures were maintained for 7 d before treatment and routinely observed under a phase-contrast inverted microscope. To quantify the neuron percentage in our cultures, we immunostained the neurons against RBFOX3/NeuN. We found that the neuron percentage was approximately 85–90%.

**Oxygen glucose-deprivation/reperfusion (OGD/R) procedures**

For OGD treatment, cells were rinsed once with warmed glucose-free DMEM and then refreshed with with O_2_- and glucose-free DMEM (Gibco), (pre-balanced in an O_2_-free chamber at 37 °C). Cells were then immediately placed in a sealed chamber (MIC-101, Billups-Rothenburg) loaded with a mixed gas containing 5% CO_2_ and 95% N_2_ for 6 min at 25 L/min. The sealed chambers were then incubated at 37 ^0^C for 4 h and 1.5 h. Reperfusion was performed by refreshing the culture with normal medium. Control cells were given equal refreshment but were incubated in glucose-containing DMEM at 37°C in an atmosphere of 5% CO_2_.

**Western blot analysis**

N2a cells, primary cultured neurons after 6 h of reperfusion and brain tissues after 12 h of reperfusion were homogenized in RIPA buffer (20 mmol/L Tris-HCl, pH 7.5, 150 mmol/L NaCl, 1 mmol/L EDTA, 1% Triton X-100, 0.5% sodium deoxycholate (Sigma, 30970), and 2% Protease Inhibitor Cocktail Tablets (Roche, 04693132001). An aliquot of 60 µg protein from each sample was separated using SDS-PAGE and transferred to a nitrocellulose membrane, which was then blocked with 5% nonfat milk in PBS (pH 7.4). The membrane was incubated with primary antibodies against LC3 (1:1,000 Sigma, L7543), p62 (1:1,000; Cell Signaling Technology, 5114), COX4I1 (1:1,000; Cell Signaling Technology, 4844S), TIMM23 (1:200; Anbo Biotechnology, C16678) and GAPDH (1:3,000; KangChen, KC-5G4) at 4 C overnight.

Secondary antibodies against rabbit (IRDye 800-coupled, 1:10,000; LI-COR, 926–32211) or mouse IgG (IRDye 700-coupled, 1:5,000; LI-COR, 926–68070) were incubated for 2 h at room temperature. Blots were visualized using an Odyssey infrared imaging system (LI-COR Biosciences, 9120) and analyzed using the Odyssey software. The relative optical density was obtained by comparing the measured values with the mean values from the control group.
